# Elucidation of the Hemostatic and Anti‐Inflammatory Effects of Walnut Shell Ethanol Extract by Network Pharmacology and Experimental Verification

**DOI:** 10.1002/fsn3.70726

**Published:** 2025-08-05

**Authors:** Ying He, Wenhui Zhao, Yanting Sun, Lei Ma, Censhu Li, Xueying Zheng, Zeguo Feng, Hui Guo, Liguo Qin, Yali Zhang

**Affiliations:** ^1^ Center for Mitochondrial Biology and Medicine, The Key Laboratory of Biomedical Information Engineering of Ministry of Education, School of Life Science Xi'an Jiao Tong University China; ^2^ Department of Endocrinology, First Affiliated Hospital of Medical College Xi'an Jiao Tong University Xi'an China; ^3^ Key Laboratory of Education Ministry for Modern Design and Rotor‐Bearing System, Institute of Design Science and Basic Components, School of Mechanical Engineering Xi'an Jiao Tong University People's Republic of China

**Keywords:** anti‐inflammation, hemostasis, LPS‐induced RAW264.7 cells, molecular docking, network pharmacology, walnut shell

## Abstract

Effective hemostatic and anti‐inflammatory measures are essential for wound healing. In this study, Ultra‐performance liquid chromatography (UPLC) combined with network pharmacology was used to analyze the composition of walnut shell ethanol extract (WSEE), and in vivo and in vitro experiments elucidated its potential mechanisms of hemostatic and anti‐inflammatory effects. The results showed that 45 components were identified from WSEE, and the NF‐κB signaling pathway, IL‐17 signaling pathway, etc., could play significant roles in this context. In vitro, WSEE exhibited procoagulant activity including shortened serum APTT (activated partial thromboplastin time, 40.4 ± 3.05 s), TT (thrombin time, 26.13 ± 1.6 s), and PT (prothrombin time, 14.6 ± 2.1 s) (*p* < 0.01), and increasing FIB levels (Fibrinogen, 3.7 ± 1.01 s). In vivo, WSEE significantly shortened the clotting time in the WSEE group (05:26 s) compared with the control group (13:25 s). Comparing to the lipopolysaccharide alone treatment group, the expression of pro‐inflammatory factors such as TNF‐α (61.63%, 73.06%, and 66.03%), IL‐1β (94.78%, 95.40%, and 88.99%), and IL‐6 (91.37%, 97.02%, and 89.00%) were inhibited at the corresponding concentrations (10, 30, and 50 mg/mL) in RAW264.7 cells stimulated by lipopolysaccharide. Moreover, ADRB2 and PTPN2 were the key target proteins associated with bleeding‐related and inflammatory‐related diseases, which were obtained from the results of network pharmacology analysis. WSEE also alleviated the expression of ADRB2 (50.53%, 56.11%, and 83.41%) and PTPN2 (95.87%, 96.56%, and 91.49%) in RAW264.7 cells stimulated by lipopolysaccharide, findings that align with the results of network pharmacology. These findings provided evidence for considering WSEE as a promising candidate for wound healing.

## Graphical abstract

Forty‐five components of WSEE were identified by UPLC‐Q‐TOF‐MS. The main targets of WSEE were PTPN2, ADRB2, LTA4H, and so on in wound healing. WSEE has an anti‐inflammatory effect in LPS‐stimulated RAW264.7 cells, procoagulant activity in vitro, and shortens hemostasis time in vivo.

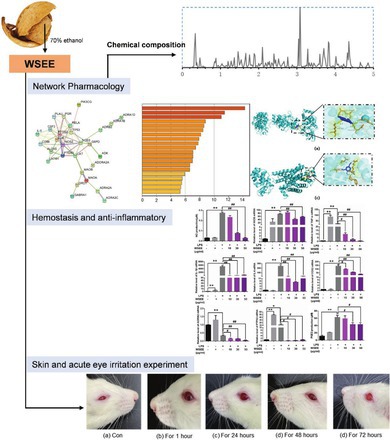

## 1 Introduction

Wound healing is a complex and dynamic process typically divided into four stages: hemostasis, inflammation, tissue formation, and remodeling. Each stage is highly coordinated with the others (Lindley et al. 2016). Upon skin injury, the tissue isolates the affected area from the environment by forming clots to prevent excessive bleeding. In this initial phase, known as hemostasis, the coagulation cascade is activated. Platelets adhere to extracellular matrix (ECM) components while releasing granular contents (Almadani et al. 2021). The primary objective during the inflammatory stage is to prevent pathogen entry while avoiding infections or more severe complications. During this phase, immune cells such as neutrophils, monocytes, and lymphocytes infiltrate the affected area. Elevated levels of pro‐inflammatory mediators are readily detectable; these mediators play a crucial role in recruiting additional immune cells from peripheral sites (Miao et al. 2024). Under physiological conditions, during the phases of wound hemostasis and inflammation, macrophages accumulate at the injury site to engulf bacteria and damaged tissues, typically within 72 h (Eming et al. 2014). In pathological conditions, however, tissue repair processes are hindered, leading to delayed wound healing, which is primarily characterized by prolonged inflammatory responses. The presence of a significant number of neutrophils and macrophages at the wound site adversely affects the expression of endogenous growth factors, restricts endothelial cell proliferation, results in inadequate angiogenesis, delays subsequent re‐epithelialization of tissues, and hinders keratinocyte formation as well as protein synthesis (Krzyszczyk et al. 2018). Additionally, it impedes the migration and proliferation of fibroblasts (Bussone 2017; Talbott et al. 2022). Therefore, implementing effective hemostatic and anti‐inflammatory measures in wound management is crucial for facilitating tissue repair.

Inflammation is an important phase of the wound healing process. In normal wound healing, anti‐inflammatory factors are one of the essential key steps; there are the following reasons: The inflammatory response can trigger the recruitment of mast cells and leukocytes to the damaged site, and then an increased uptake of oxygen, which is called “respiratory burst” causes the release of ROS (reactive oxygen species) from leukocytes, including activated macrophages (Schafer and Werner 2008). Meanwhile, excessive ROS production can also lead to sustained pro‐inflammatory cytokine secretion and induction of matrix metalloproteinases, which have detrimental effects on wound healing through directly and indirectly modifying and/or degrading ECM proteins, impairing the function and cell migration of dermal fibroblasts and keratinocytes (Yahaya et al. 2018). Excessive ROS production also leads to oxidative stress, which can cause injury to all vital cellular components such as DNA, proteins, and membrane lipids, ultimately triggering cell death and affecting wound healing (Wagener et al. 2013). In addition, ROS may activate NF‐κB signal transduction pathways, which are classical inflammatory pathways, driving the expression of various inflammatory cytokines such as tumor necrosis factor (TNF)‐α, interleukin (IL)‐1β, and IL‐6 (He et al. 2022; Zhang, Zhang, et al. 2023). Pro‐inflammatory factors also play a crucial role in the process of wound healing, initiating and coordinating the complex healing cascade; however, they must be precisely regulated to prevent excessive or prolonged inflammatory responses that could lead to tissue damage and delayed healing. After tissue injury, damaged cells, platelets, and local immune cells (such as mast cells and macrophages) rapidly release a variety of pro‐inflammatory factors, including TNF‐α, IL‐1β, IL‐6, and IL‐8 (Krzyszczyk et al. 2018). TNF‐α, IL‐1β, and IL‐6 can increase the permeability of the vessel walls, allowing plasma proteins (such as fibrinogen, complement, antibodies) and immune cells to seep out of the blood vessels and enter the wound area (Zheng et al. 2013). TNF‐α and IL‐1β also activate the arriving immune cells, including neutrophils and macrophages, enhancing their phagocytic ability and bactericidal activity (Saia et al. 2015). IL‐8 is a kind of chemokine that can draw neutrophils, differentiated macrophages, and lymphocytes from the circulation to the wound site (Zhang, Jiang, et al. 2023). In addition, pro‐inflammatory factors can trigger the proliferative phase of wound healing. Pro‐inflammatory factors are necessary for wound healing, but their excessive or prolonged presence is one of the main causes of chronic wound formation, delayed healing, and pathological scarring; thus, regulating the balance of pro‐inflammatory factor secretion is also key to promoting wound healing (Almadani et al. 2021).

In recent years, derivatives of natural products have attracted much more attention due to their relatively low toxicity and high bioactivities (Wu et al. 2016). Traditional Chinese medicine has a long‐standing history and extensive applications in trauma repair (Zhou et al. 2024). Its advantages include multicomponent formulations that target various pathways while remaining cost‐effective, safe, and reliable. The active ingredients in Chinese medicine significantly contribute to trauma repair by regulating endogenous growth factors, binding to receptors, and activating downstream signaling pathways (Ning et al. 2022; Zhou et al. 2022). Modern pharmacological research has identified several active components with therapeutic potential such as Sanqi saponins Ft1, Sanqi saponins R1, ginsenoside Rg1 (Zhang et al. 2024), Baiji (Zeng et al. 2023), curcumin obtained (Abd El‐Hack et al. 2021), α‐frankincense acid source (Efferth and Oesch 2022), myrrh steroid ketone (Suliman et al. 2022), blood dragon extract (including perchlorate) (Krishnaraj et al. 2019), and Danshen extract (which contains salvianolic acid B and other compounds) have been recognized for their analgesic, anti‐inflammatory, and tissue‐regenerative properties (Mahalakshmi et al. 2021; Yuen et al. 2021).

As a common natural product, walnut is an arboreal plant belonging to the *Juglan Linn*, *Carya*, or *Annamocarya* within the *Juglandaceae* family (Liu et al. 2019; Zhang et al. 2020) and is widely distributed across the globe. Compared with other natural products or synthetic agents (such as Sanqi and Yunnan Bai Yao), the cost of WSEE is significantly lower; Walnuts are widely available and are cultivated in various regions of China, and WSEE are also easily obtained. The walnut shell, which is the endocarp of the ripe fruit, comprises approximately 30% of total walnut production but is often discarded or incinerated. This practice contributes to environmental pollution and represents a significant waste of resources. Walnut shells primarily contain phenolic acids, saponins, flavonoids, esters, and polysaccharides among other chemical constituents that possess antioxidant and antibacterial properties (Caldas et al. 2019; Silveira et al. 2020).

Due to the complex composition and multiple targets of walnut shell (Hu et al. 2019; Jahanban‐Esfahlan and Amarowicz 2018), elucidating the characteristics of its multicomponent, multitarget, and multilink functions is one of the bottleneck problems at present. Combined with computer science, molecular biology, and pharmacy, network pharmacology can fully elucidate the multicomponent, multitarget, and multilink functional characteristics of traditional Chinese medicine (Li et al. 2014; Liang et al. 2014; Zhou et al. 2021; Zuo et al. 2018).

In this study, we hypothesize that the WSEE exhibits procoagulant activity in vivo, and may exert anti‐inflammatory effects through inhibiting the secretion of pro‐inflammatory factors in vitro. Meanwhile, network pharmacology analysis may hint at the related pathways (such as cGMP‐PKG signaling pathway, IL‐17 signaling pathway, and NF‐κB signaling pathway) of the anti‐inflammatory effect in WSEE. Ultra‐performance liquid chromatography (UPLC) combined with network pharmacology was employed to analyze the chemical constituents, potential active components, targets, and mechanisms associated with walnut shell ethanol extract (WSEE) in relation to procoagulant activity and anti‐inflammation effects. The molecular docking technique was utilized to investigate the specificity and potential for anti‐inflammatory effects of WSEE. The mechanisms involved in hemostasis and anti‐inflammation were elucidated through both in vivo and in vitro experiments, providing a theoretical foundation for the clinical studies and exploration of potential anti‐inflammatory drugs.

## 2 Materials and Methods

### 2.1 Preparation of WSEE

Walnut shells were purchased from Xi'an, Shaanxi, China in June 2023, with identification conducted by Prof. Yali Zhang from Xi'an Jiaotong University. Crushed walnut shells were soaked in 70% ethanol at a final ratio of 1:30 for 24 h before being filtered through a 75 μm filter. The resulting liquid was collected twice. Subsequently, the aqueous extract was concentrated to its original volume through heat evaporation according to the previous study (Blasi et al. 2022).

### 2.2 UPLC‑MS/MS Analysis of WSEE

The chemical composition analysis was performed using ultra‐performance liquid chromatography coupled with quadrupole time‐of‐flight mass spectrometry (Type: WATERS I‐Class VION IMS QTof) (Salem et al. 2022). Samples were diluted 10‐fold with methanol prior to centrifugation at 20,000 rpm for 10 min at 4°C. The supernatant was then collected and filtered through a 0.22 μm membrane prior to detection.

The samples were separated using the UHPLC on a Waters ACQ chromatographic column. The reaction conditions included a column temperature of 40°C, a flow rate of 0.4 m/min, and a sample volume of 10 μL. A mobile phase consisting of 0.1% formic acid in water (Solution A) and acetonitrile (Solution B) was employed, following the gradient program: 5.0%–13% B from 0 to 6 min, 13% B from 6 to 20 min, 13%–22% B from 20 to 60 min, and 22.0%–100.0% B from 60 to 90 min. The electrospray ionization (ESI) source was operated in a positive mode.

For the establishment of HPLC fingerprinting for the WSEE, samples were analyzed under specific chromatographic conditions, resulting in a liquid phase map for extracts derived from walnut shell. The software Similarity Evaluation System for Chromatographic Fingerprint of Traditional Chinese Medicine (version 2012) was utilized to obtain the fingerprint map of WSEE.

### 2.3 Prediction of the Bioactive Ingredient Targets

SMILES representations of bioactive ingredients were obtained from the PubChem database (https://pubchem.ncbi.nlm.nih.gov/). These SMILES notations were subsequently imported into the Swiss Target Prediction platform (http://www.swisstarget‐prediction.ch/) to identify potential targets for these bioactive compounds. According to the previous method (Lussi et al. 2023), using “Periodontitis” as a keyword, we uploaded the identified targets to the UniProt database (https://www.uniprot.org/uploadlists) to ascertain the key bioactive components. This process facilitated a comparison against inflammation‐related and bleeding‐related targets for screening potential therapeutic candidates.

### 2.4 Construction of Targets Disease Network

Protein–protein interactions (PPIs) among target proteins associated with bioactive ingredients and diseases were compiled using STRING (https://string‐db.org/, version 11.0), a database that integrates known and predicted PPIs through bioinformatic methodologies (Szklarczyk et al. 2021). In this study, we focused exclusively on “ *Homo sapien* ”, collecting the PPIs with confidence scores exceeding 0.4 while excluding disconnected nodes from the network.

### 2.5 GO and KEGG Enrichment Analysis

The selected target points underwent pathway enrichment analysis using Kyoto Encyclopedia of Genes and Genomes (KEGG), along with Gene Ontology (GO) biological process analysis (Gao et al. 2023), utilizing the DAVID database (https://david.ncifcrf.gov/).

The signaling pathways closely associated with the targets were imported into Cytoscape 3.6.1 software to construct a network diagram.

### 2.6 Molecular Docking

The top four disease‐associated targets were identified from the compound–target–pathway network based on their node degree values. The 3D structures of these target proteins were obtained from the RCSB PDB database. Nonreceptor ligands and water molecules were removed using PyMOL (http://www.pymol.org/pymol), and the refined structures were saved in PDBQT format. The 2D structures of the bioactive compounds were retrieved from the PubChem database. ChemBio3D Ultra 14.0 was employed to minimize the free energy of each ligand, ensuring conformational stability, and the optimized structures were converted to PDBQT format. Molecular docking was performed using AutoDock Vina with its default scoring function, which integrates steric, hydrophobic, hydrogen bonding, and torsional entropy terms to estimate binding affinity.

### 2.7 The Anti‐Inflammatory Effect of WSEE on RAW 264.7 Cells

#### 2.7.1 Proliferation Activity Assay

Macrophage RAW264.7 cells (ATCC TIB‐71) were seeded in 96‐well plates with a concentration of 2 × 10^4^ cells/mL during their logarithmic growth phase and treated with various concentrations of WSEE (1, 2, 3, 10, 30, 50, and 100 μg/mL). After a 24 h incubation period, 100 μL of MTT solution (0.5 mg/mL) (CAS: 298–93‐1, Sangon Biotech Bioengineering, China) was added to each well. Following an additional incubation of 4 h, the cell supernatants were discarded and replaced with 150 μl of DMSO (CAS: 67–68‐5, Sangon Biotech Bioengineering, China) for cell lysis and crystal solubilization. Absorbance was recorded at 490 nm. Three parallel wells were established for each group, and the experiments were repeated three times to ensure the accuracy of the results.

#### 2.7.2 Phagocytosis Assay

Macrophage RAW 264.7 cells were seeded in 96‐well plates at a density of 2 × 10^5^ cells/mL and treated with various final concentrations of WSEE (10, 30, and 35 μg/mL). LPS (CAS: L2880, Sigma, USA) at a concentration of 1 μg/mL served as the positive control. After incubation for 48 h at 37°C in a 5% CO_2_ atmosphere, the medium was discarded and replaced with 50 μL of a 0.1% neutral red dye solution (CAS: G1318, Solarbio, China) per well according to the previous study (Wang et al. 2019). Following a 3 h incubation period, the plates were washed twice with phosphate‐buffered saline (PBS). Each well then received 50 μL of cell lysate (a mixture of ethanol and acetic acid in equal volumes), which was gently shaken for 10 min until fully blended. Absorbance readings were taken at 540 nm.

#### 2.7.3 NO Production

Macrophage RAW 264.7 cells were seeded in a 24‐well plate at a density of 2 × 10^5^ cells/mL and treated with various concentrations of WSEE (10, 30, and 50 μg/mL) for 48 h, as described in Section 2.6. LPS was used as a positive control at a concentration of 1 μg/mL. Referring to relevant literature (Cao et al. 2019), the level of NO secretion was measured using commercial kits (CAS:bc4960, Solarbio, China) according to the manufacturer's instructions.

#### 2.7.4 Real‐Time PCR Analysis

RAW264.7 cells were subsequently plated in 6‐well plates at a concentration of 1 × 10^6^ cells/ml and cultured in a CO_2_ incubator for 24 h. The cells were treated as described in Section 2.7.2. Total RNA from RAW264.7 cells was isolated using Trizol (CAS: T9424, Sigma, USA) and reverse transcribed into first‐strand cDNA using the Easy Script First‐Strand cDNA Synthesis Super Mix kit (CAS: AG11706, AG, China). The mRNA levels of iNOS, TNF‐α, IL‐6, and IL‐10 were quantified by real‐time PCR employing gene‐specific primers. The expression levels of the target genes were normalized against GAPDH as an internal control. Real‐time PCR was performed on a BIO‐RAD CFX Connect Real‐Time PCR System (BIO‐RAD, USA) using the Pro Taq HS Premix Probe qPCR Kit II (CAS: AG11702, AG, China). The amplification conditions included one cycle at 95°C for 30 s, followed by 40 cycles consisting of denaturation at 95°C for 5 s and annealing/extension at 60°C for 30 s. The quantitative primers in mice are listed in Table 1.

**TABLE 1:** Sequences of oligonucleotide primers for qRT‐PCR (mouse).

Target	Forward (5′‐3′)	Reverse (5′‐3′)	Product size	References
GAPDH (mouse)	AAGAGCACAAGAGGAAGAGAGAGAC	GTCTACATGGCAACTGTGAGGAG	103 bp	(Hou et al. 2019)
IL‐1β (mouse)	ATGGCAACTGTTCCTGAACTCAACT	CAGGACAGGTATAGATTCTTTCCTT	563 bp	(Surayot et al. 2014)
IL‐6 (mouse)	TTCCTCTCTGCAAGAGACT	TGTATCTCTCTGAAGGACT	432 bp	(Yang et al. 2019)
iNOS (mouse)	GGAATCTTGGAGCGAGTTGT	GCAGCCTCTTGTCTTTGACC	103 bp	(Takara et al. 2024)
COX‐2 (mouse)	CCACTTCAAGGGAGTCTGGA	AAGTAGGTGGACTGTCAATC	110 bp	(Bi et al. 2019)
ADRB2 (mouse)	ATGTCGGTTATCGTCCTGGC	GGTTTGTAGTCGCTCGAACTTG	81 bp	(Tiwari et al. 2023)
PTPN2 (mouse)	ATGTCGGCAACCATCGAGC	TGCAGTTTAACACGACTGTGAT	185 bp	(Zhang, Zhang, et al. 2023)

#### 2.7.5 The Procoagulant Activity of WSEE In Vivo and In Vitro

All animal experiments were carried out in accordance with Guidance on the operation of the Animals (Scientific Procedures) Act 1986 and associated guidelines, EU Directive 2010/63 for the protection of animals used for scientific purposes, and the guidelines of the Biomedical Ethics Committee of the Health Science Center of Xi'an Jiao Tong University (approval number: 2022 (293) 0.22 (293)). The sex of the animals did not influence the results of the study. The method of this part was according to the recent study with a few modifications (Liang et al. 2023). Twelve healthy male rats aged 8 weeks were purchased from the Model Animal Research Center of Xi'an Jiao Tong University (Xi'an, China) and randomly divided into three groups with four rats in each group. The three groups were: Blank control group with normal saline; positive control group with Yunnan Bai Yao; and administration group with WSEE, respectively. Taking several medical cotton balls, the quality of each medical cotton ball was the same as the current size; 0.25 g WSEE and 30 mg Yunnan Bai Yao were plated on the medical cotton ball, respectively, and the rest of the cotton balls were soaked in 1 mL normal saline. The rats were anesthetized, and the rat tail, about 1 cm was cut off away from the tip of the rat tail until the blood could flow out smoothly. After the bleeding model was established, the cotton ball was applied to the wound immediately, and the time was recorded. The wound was opened every 30 s to observe the bleeding situation until there was no obvious large amount of blood flow from the wound within 15–20 s, and then the wound was considered to have stopped bleeding, and the time was recorded.

All human experiments were performed with the Guidelines of the Biomedical Ethics Committee of Health Science Center of Xi'an Jiao Tong University (Ethics No. 2021272). The coagulation procedure in vitro is as follows: Blood samples from 30 healthy volunteers were collected into vials containing a sodium citrate solution (9:1). Following collection, the samples were subjected to centrifugation at 3000 rpm for 30 min to obtain supernatants, which were the plasma. The plasma was divided into three groups, including a blank control group with normal saline (500 μL) positive control group with Yunnan Bai Yao (10 mg), and an administration group with WSEE (0.1 g). The mixed plasma was then analyzed for activated partial thromboplastin time (APTT), prothrombin time (PT), thrombin time (TT), and fibrinogen (FIB) levels, in accordance with the specifications provided by each respective assay kit (20,172,400,178, Zhongtai biotech, China).

#### 2.7.6 Skin Allergy Test and Acute Eye Allergy Test in Rats

All animal experiments were carried out in accordance with Guidance on the operation of the Animals (Scientific Procedures) Act 1986 and associated guidelines, EU Directive 2010/63 for the protection of animals used for scientific purposes, and the guidelines of the Biomedical Ethics Committee of the Health Science Center of Xi'an Jiao Tong University (approval number: 2022 (293) 0.22 (293)). According to the recent study (Ortega et al. 2016), five healthy rats were acclimated for 3 days prior to the experiment. The fur on both sides of their spines was carefully trimmed without damaging the epidermis. After a 24 h period, 0.5 mL of 1 mg/mL of WSEE of the test solution was uniformly applied to the depilated area, which measured 2 × 2 cm, while 0.5 mL of distilled water served as a control. The rats were then returned to their normal feeding routine once the test solution had been fully absorbed. The test solution was administered once daily for a duration of 14 days. Starting on the second day, the fur in the application area was trimmed before each application, and any residual extract was removed with distilled water. After 1 h, observations and photographs of the rat skin were taken.

Five healthy rats were selected and acclimated for 3 days. Both eyes of each rat were examined 1 day prior to testing. Rats exhibiting symptoms such as eye allergy, corneal defects, or conjunctival damage were excluded from participation (Arteaga et al. 2014).

The lower eyelid of one eye was gently retracted, allowing approximately 0.1 mL of 1 mg/mL of WSEE extract to be instilled into the conjunctival sac, and 0.1 mL of distilled water served as the negative control. This prompted passive closure of both the upper and lower eyelids for 1 s to minimize the loss of the extract. The contralateral eye remained untreated as a control. After 30 s, both eyes were rinsed with distilled water for at least 30 s. Examination of the cornea, iris, and conjunctiva occurred at intervals post‐instillation: at 1, 24, 48, and 72 h later. If no adverse response occurred by 72 h posttreatment, testing would conclude.

### 2.8 Statistical Analysis

Data analysis was conducted using GraphPad Prism version 7.0 software for annotation purposes. Statistical analyses included *t*‐tests and one‐way analysis of variance (ANOVA) as appropriate for comparisons between two or more groups, respectively. Statistical significance was determined using a *p*‐value threshold of less than 0.05.

## 3 Results and Discussion

### 3.1 The Chemical Composition of WSEE

**FIGURE 1:**
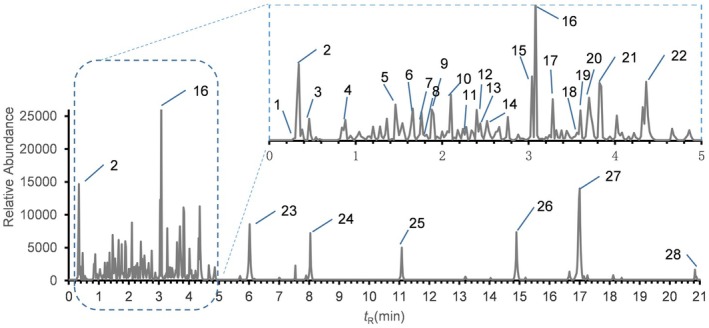
Total ion chromatogram of UPLC‐Q‐TOP‐MS of WSEE.

A UPLC‐Q‐TOP‐MS protocol was employed to analyze the chemical composition of WSEE. The total ion current is illustrated in Figure 1. Based on the compound m/z information and comparisons with the literature, a total of 45 known compounds were identified (Table S1), with 27 chemical components exhibiting a content greater than 1%, as presented in Table 2.

**TABLE 2:** Chemical constituents of WSEE (relative abundance higher than 1%).

No.	Compound name	CAS No.	Molecular formula	*m/z* [M‐H]	t_R_/min	Relative abundance (%)
1	Paeonoside A		C_14_H_18_O_9_	353.0841	0.32	3.17
2	Dihydrocarthiopanate‐4′‐O‐β‐D‐glucopyranoside		C_21_H_32_O_1_	483.1635	0.34	6.70
3	Roseoside	54835‐70‐0	C_19_H_30_O_8_	409.1861	0.46	1.66
4	4‐Hydroxybenzoic acid	99‐96‐7	C_7_H_6_O_3_	139.0391	0.89	1.58
5	4′‐Hydroxy‐3′‐methoxyacetophenone	498‐02‐2	C_9_H_10_O_3_	167.0703	1.53	1.33
6	3,4,5‐Trihydroxybenzoic acid (gallic acid)	149‐91‐7	C7H6O5	169.0497	1.65	2.11
7	3‐Hydroxy‐1‐(4‐hydroxy‐3‐methoxyphenyl) propan‐1‐one	2196‐18‐1	C_10_H_12_O_4_	197.0809	1.76	1.70
8	2‐Aldehyde‐3‐hydroxyphenylpropanol			167.0702	1.88	2.36
9	4‐Hydroxybenzaldehyde	123‐08‐0	C_7_H_6_O_2_	123.0443	1.9	2.02
10	Juglanoside A		C_16_H_20_O_7_	347.1126	2.1	3.48
11	3‐Hydroxy‐5‐methoxybenzaldehyde	57179‐35‐8	C_8_H_8_O_3_	153.0547	2.28	1.06
12	Ethyl vanillate	617‐05‐0	C_10_H_12_O_4_	197.081	2.4	2.34
13	3,5‐Dimethoxy‐4‐hydroxybenzaldehyde (syringaldehyde)	134‐96‐3	C_9_H_10_O	183.0652	2.42	1.32
14	5‐Hydroxy‐1,4‐naphthoquinone (juglone)	481‐39‐0	C_10_H_6_O_3_	174.15	2.51	1.50
15	1‐ (4′‐Hydroxyphenyl)‐allyl aldehyde	2538‐87‐6	C_9_H_8_O_2_	149.06	3.06	4.86
16	3‐(4‐Hydroxy‐3‐methoxyphenyl)‐2‐propenal (coniferaldehyde)	458‐36‐6	C_10_H_10_O_3_	179.0702	3.08	11.41
17	Guaiacol	90‐05‐1	C_7_H_8_O_2_	147.0443	3.28	3.15
18	3‐(2,5‐Dimethoxyphenyl) propionic acid	10538‐49‐5	C_11_H_14_O_4_	211.0962	3.61	2.31
19	2‐Methoxy‐4‐vinylphenol	7786‐61‐0	C_9_H_10_O_2_	151.0753	3.68	1.87
20	N‐Octylmalonic acid	760‐55‐4	C_11_H_20_O_4_	255.1013	3.7	3.26
21	Phenol acetate	122‐79‐2	C_8_H_8_O_2_	137.0598	3.83	10.43
22	4‐Allyl‐2‐methoxy‐pheno (eugenol)	97‐53‐0	C_10_H_12_O_2_	164.0863	4.36	4.46
23	Campestrol	4651‐51‐8	C_28_H_48_O	439.3312	6.03	3.39
24	Pyrogallol	87‐66‐1	C_6_H_6_O_3_	149.023	8.03	2.89
25	1–2‐Benzenediol (catechol)	52936‐64‐8	C_30_H_48_O_5_	489.3574	11.07	1.98
26	β‐Sitosterol	83–46‐5	C_29_H_50_O	437.3732	14.89	2.91
27	4‐Stigmasten‐3‐one	1058‐61‐3	C_29_H_48_O	413.3776	17	6.14

Inflammation is a crucial stage in the wound healing process; however, continuous inflammation will delay the healing process by damaging the growing tissue of the repair site. Meanwhile, an excessive inflammatory response will lead to oxidative stress, which produces a large number of oxidative intermediates, further exacerbating the difficulty of wound healing (Almadani et al. 2021). Bacterial infection is also a common cause of delayed wound healing. If not timely controlled, it may also lead to systemic infection and even be life‐threatening (Lu et al. 2023). The main chemical components of WSEE include phenolic acids, aldehydes, and ketones. Studies have shown that phenolic acids can inhibit the phosphorylation of IKKα/β and IκBα to inhibit the activation of the NF‐κB pathway (Xie et al. 2024); phenolic acids can also inhibit the secretion of IL‐17 and block the combination of IL‐17A and IL‐17RA to improve skin inflammation in psoriatic mice (Lo et al. 2019); vanillin attenuates pro‐inflammatory factors in a tMCAO mouse model via the inhibition of phosphorylated NF‐κB (Wang et al. 2022); ketone compounds such as isoliquiritigenin can downregulate the LPS‐induced inflammatory response in MACT cells by inhibiting the phosphorylation levels of NF‐κB p65 and blocking the nuclear transfer of NF‐κB p65 (Li et al. 2022).

### 3.2 Network Pharmacology

#### 3.2.1 Building Candidate Compounds Library of WSEE

Information regarding the compounds present in WSEE and their potential targets was sourced from traditional Chinese medicine databases, including TCMSP, TCMID, and BATMAN‐TCM. The compounds were screened based on ADME parameters, such as oral bioavailability (OB ≥ 30%) and drug‐likeness (DL ≥ 0.18) within the TCMSP database.

Compounds that adhere to the five rules of drug‐like properties demonstrate enhanced pharmacokinetic characteristics and bioavailability during in vivo metabolic processes, making them more likely candidates for development into oral medications. The five criteria are as follows: (1) molecular weight < 500; (2) hydrogen bond acceptors < 5; (3) hydrogen bond donors < 10; (4) oil–water partition coefficient < 5; and (5) the number of rotatable bonds ≤ 10.

**FIGURE 2:**
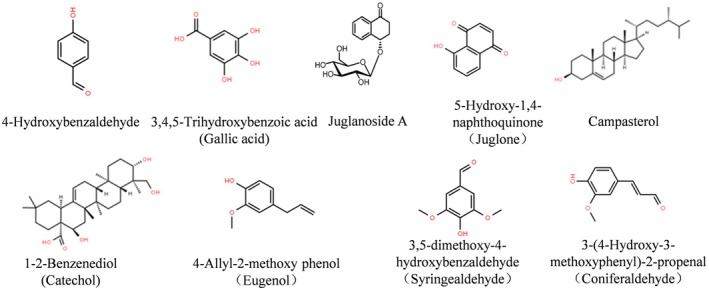
Structural formula of candidate compounds.

The effective chemical compositions identified in WSEE are summarized in Table 3, based on the five established criteria. Seven compounds that fulfill all five criteria for drug‐like properties include: 4‐hydroxybenzaldehyde, 3,4,5‐trihydroxybenzoic acid (gallic acid), juglanoside A, 5‐hydroxy‐1,4‐naphthoquinone (juglone), campestrol, 1,2‐benzenediol (catechol), 4‐allyl‐2‐methoxy‐phenol (eugenol), 3,5‐dimethoxy‐4‐hydroxybenzaldehyde (syringaldehyde), and 3‐(4‐hydroxy‐3‐methoxyphenyl)‐2‐propenal (coniferaldehyde). The structural formulas of these candidate compounds are illustrated in Figure 2. Based on their composition and functional characteristics, juglanoside A and juglone were categorized together, while eugenol and syringaldehyde formed another group, resulting in a total of seven categories for further analysis.

**TABLE 3:** Candidate compounds library.

No	Compound name	Pub Chem CID	OB/%	Caco‐2	DL	Mw	HBD	miLogP	HBA	NROTB
1	4‐Hydroxybenzaldehyde	126	29.98	0.82	0.02	122.12	1	1.25	2	1
2	3,4,5‐Trihydroxybenzoic acid (gallic acid)	370	31.69	−0.09	0.04	170.12	4	0.59	5	1
3	Juglanoside A	/	/	/	/	324.33	4	−0.57	7	3
4	5‐Hydroxy‐1,4‐naphthoquinone (juglone)	3806	25.74	0.42	0.07	174.16	1	1.4	3	0
5	Campestrol	/	35.41	1.36	1.36	400.69	1	8.18	1	5
6	1–2‐Benzenediol (catechol)	104,361	29.74	0.82	0.02	488.71	4	4.63	5	2
7	4‐Allyl‐2‐methoxy‐pheno (Eugenol)	3314	60.62	1.1	0.32	164.2	1	2.1	2	3
8	3,5‐Dimethoxy‐4‐hydroxybenzaldehyde(Syringaldehyde)	8655	67.06	0.71	0.05	182.18	1	1.08	4	3
9	3‐(4‐Hydroxy‐3‐methoxyphenyl)‐2‐propenal (coniferaldehyde)	5352904	61.25	0.79	0.05	178.19	1	1.82	3	3

#### 3.2.2 Screen the Targets of WSEE and Construct a Network Diagram

**FIGURE 3:**
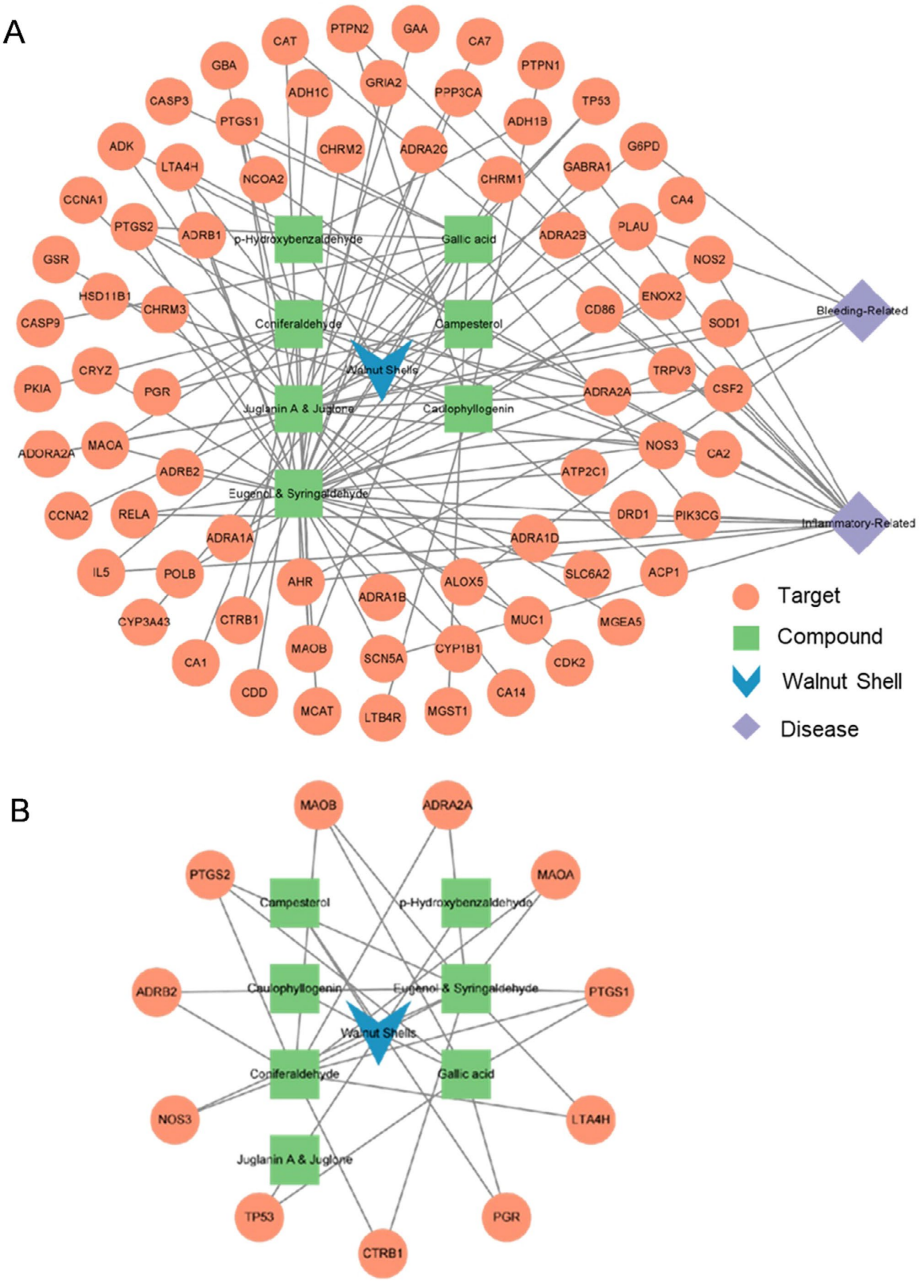
Network diagram of WSEE. (A) Candidate compounds‐target‐disease; (B) candidate compound‐key target. The purple represents disease. The blue quadrilateral represents WSEE. The green triangle represents the target. The orange circle represents the active compound contained in WSEE.

The targets within the library of candidate compounds were identified using the SysDT model. The anti‐inflammatory and coagulation‐related target genes were extracted from the OMIM and TTD databases, followed by mapping these targets with those of WSEE screened in the compound‐key target network (Figure 3A). This approach allows for an in‐depth exploration of the pharmacological effects associated with the synergistic hemostatic and anti‐inflammatory properties of walnut shells. Through this analysis, 74 potential targets corresponding to the seven active ingredients were identified.

**FIGURE 4:**
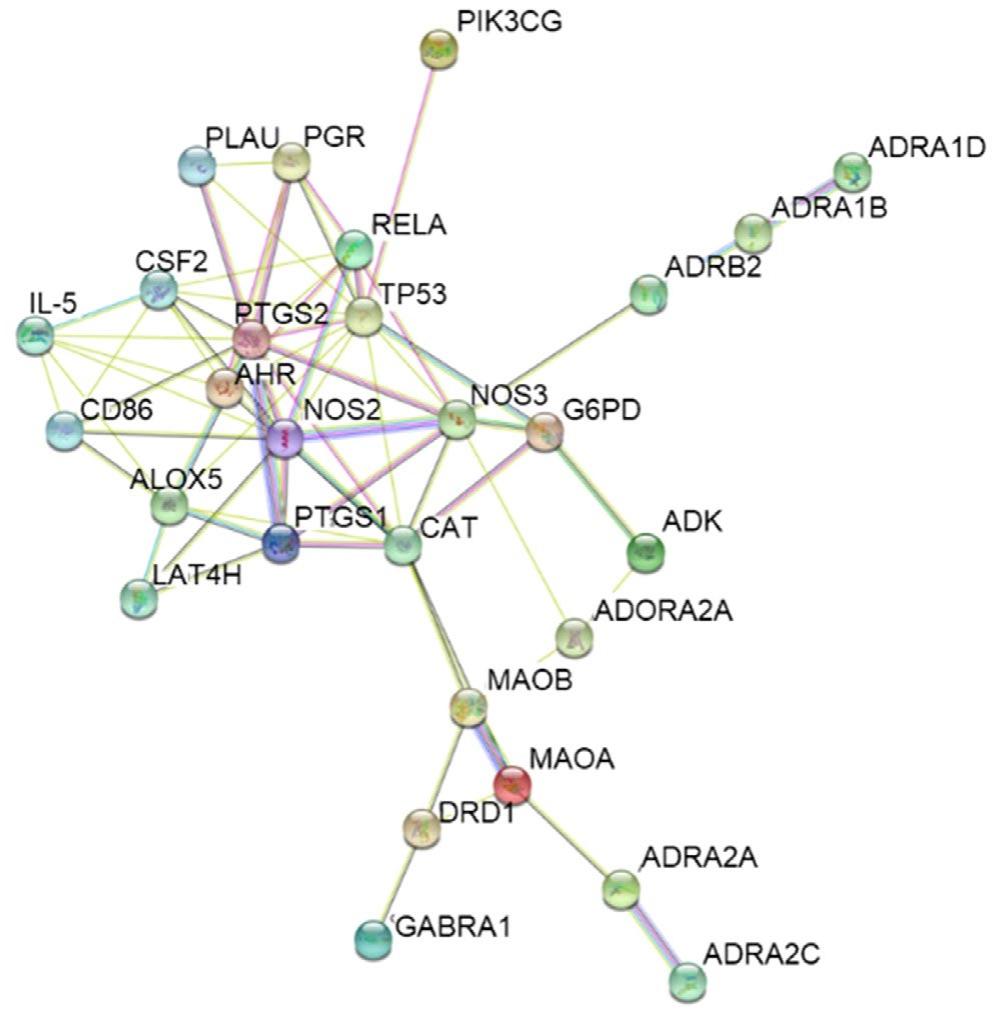
Target protein interaction diagram based on STRING.

The key targets among WSEE compounds were selected based on a degree range of 2 ≤ degree ≤ 8 within the compound‐gene network, as illustrated in Figure 3B. This analysis identified a total of 11 key targets across the seven active ingredients. The preliminary elucidation of the pharmacological effects of walnut shell was achieved through this network analysis. These key targets were subsequently imported into the STRING database to obtain data regarding protein–protein interactions (Figure 4).

#### 3.2.3 Pathway Enrichment Analysis

**FIGURE 5:**
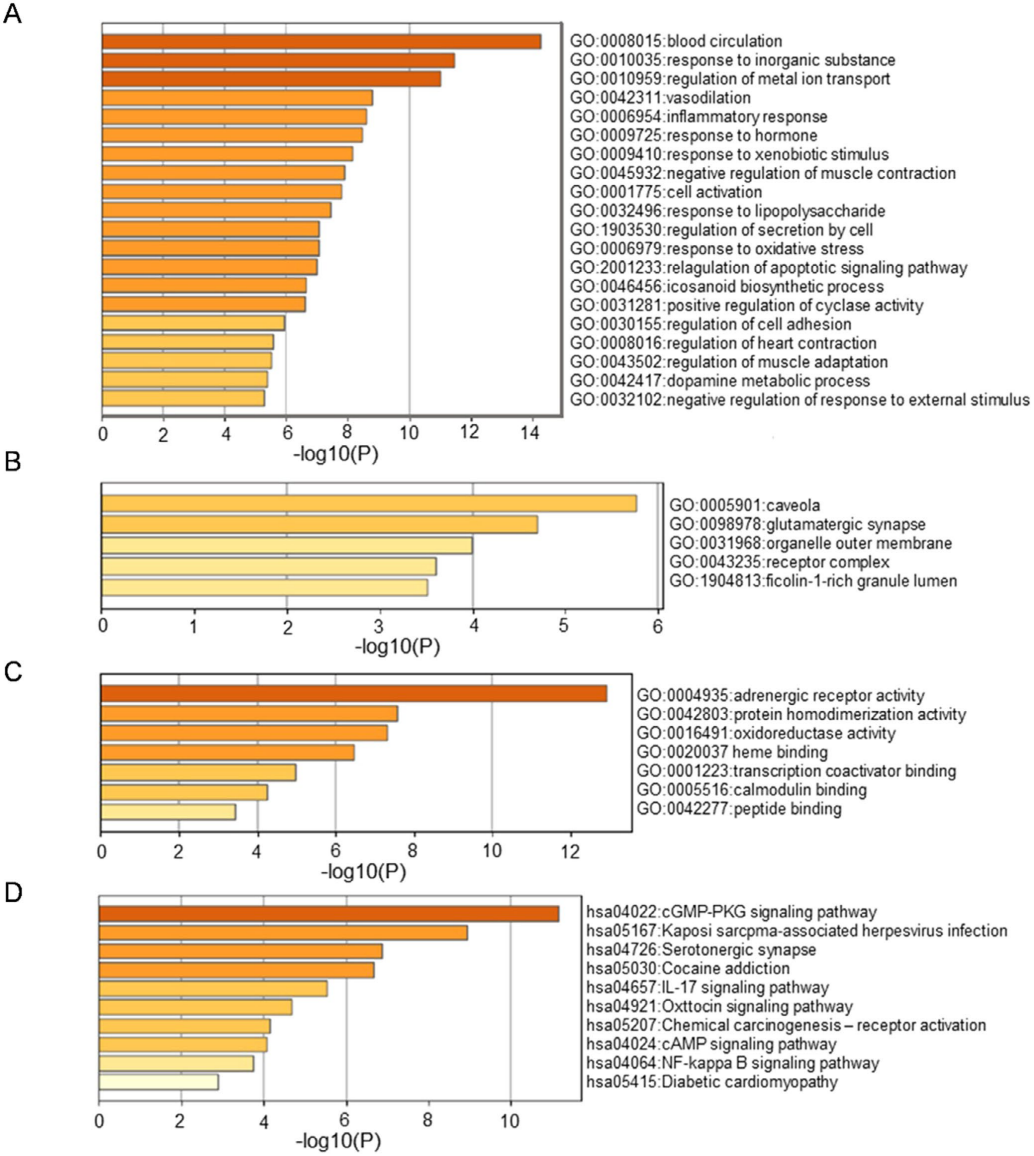
Core targets enrichment analysis map: (A) the analysis result of GO biological process; (B) the analysis result of GO cellular components; (C) the analysis result of GO molecular function; (D) the analysis result of KEGG.

The predicted core target gene information was then entered into the Metascape database to retrieve related pathway information. The parameter settings included input species: *H. sapiens* ; analysis as species: *H. sapiens* , with enrichment results displayed in Figure 5. Gene Ontology (GO) function enrichment analysis for core targets was conducted on the DAVID platform at a significance level set at *p* < 0.05, yielding a total of 389 GO items, 328 of which pertain to biological processes (BP). These processes primarily involve blood circulation, response to inorganic substances, regulation of metal ion transport, vasodilation, inflammatory response, response to hormones, and response to xenobiotic stimuli, as well as the negative regulation of muscle contraction, among others. The analysis identified 28 cellular components (CC), including caveolae, glutamatergic synapses, organelle outer membranes, receptor complexes, and ficolin‐1‐rich granule lumens. Additionally, 33 molecular functions (MF) were associated with activities such as adrenergic receptor activity, protein homodimerization activity, oxidoreductase activity, heme binding, transcription coactivator binding, calmodulin binding, and peptide binding. The top 10 enriched pathways identified through KEGG analysis included the cGMP‐PKG signaling pathway, Kaposi sarcoma‐associated herpesvirus infection, serotonergic synapse, IL‐17 signaling pathway, oxytocin signaling pathway, chemical carcinogenesis–receptor activation, cAMP signaling pathway, NF‐κB signaling pathway, and diabetic cardiomyopathy. Furthermore, relevant information regarding gene function and the pathway details of core targets derived from KEGG analysis are presented in Table 4.

**TABLE 4:** Key target information in KEGG pathway.

No.	KEGG pathway	Gene ID
1	cGMP‐PKG signaling pathway	146|147|150|152|154|4846|5294|5530
2	Calcium signaling pathway	135|146|147|154|1812|4843|4846|5530
3	Neuroactive ligand–receptor interaction	135|146|147|150|152|154|1812|2554
4	Adrenergic signaling in cardiomyocytes	146|147|154|5294|6331
5	Salivary secretion	146|147|154
6	Vascular smooth muscle contraction	135|146|147
7	Kaposi sarcoma‐associated herpesvirus infection	942|1437|5294|5530|5743|5970|7157
8	Transcriptional misregulation in cancer	942|1437|5328|5970|7157
9	Small cell lung cancer	4843|5743|5970|7157
10	Toxoplasmosis	240|4843|5294|5970
11	Leishmaniasis	4843|5743|5970
12	Lipid and atherosclerosis	4846|5530|5970|7157
13	Human T‐cell leukemia virus 1 infection	1437|5530|5970|7157
14	Human cytomegalovirus infection	5530|5743|5970|7157
15	Longevity regulating pathway	847|5970|7157
16	Pathways of neurodegeneration—multiple diseases	847|4843|5530|5743|5970
17	Prostate cancer	5328|5970|7157
18	Amoebiasis	1437|4843|5970
19	C‐type lectin receptor signaling pathway	5530|5743|5970
20	HIF‐1 signaling pathway	4843|4846|5970
21	Pathways in cancer	3567|4843|5743|5970|7157
22	Sphingolipid signaling pathway	4846|5970|7157
23	Relaxin signaling pathway	4843|4846|5970
24	Fluid shear stress and atherosclerosis	4846|5970|7157
25	PI3K‐Akt signaling pathway	4846|5294|5970|7157
26	Amyotrophic lateral sclerosis	847|4843|5530|7157
27	Cellular senescence	5530|5970|7157
28	Alzheimer disease	4843|5530|5743|5970
29	Tuberculosis	4843|5530|5970
30	Shigellosis	1437|5970|7157
31	MAPK signaling pathway	5530|5970|7157
32	Human papillomavirus infection	5743|5970|7157
33	Serotonergic synapse	240|4128|4129|5742|5743
34	Arachidonic acid metabolism	240|4048|5742|5743
35	Regulation of lipolysis in adipocytes	154|5742|5743
36	Cocaine addiction	1812|4128|4129|5970
37	Arginine and proline metabolism	4128|4129|4843|4846
38	Amphetamine addiction	1812|4128|4129|5530
39	Parkinson disease	135|1812|4128|4129|7157
40	Dopaminergic synapse	1812|4128|4129|5530
41	Tryptophan metabolism	847|4128|4129
42	Alcoholism	135|1812|4128|4129
43	IL‐17 signaling pathway	1437|3567|5743|5970
44	T cell receptor signaling pathway	1437|3567|5530|5970
45	Fc epsilon RI signaling pathway	240|1437|3567
46	Th1 and Th2 cell differentiation	3567|5530|5970
47	TNF signaling pathway	1437|5743|5970
48	JAK–STAT signaling pathway	1437|3567|5771
49	Oxytocin signaling pathway	4846|5294|5530|5743
50	VEGF signaling pathway	4846|5530|5743
51	Platelet activation	4846|5294|5742
52	Apelin signaling pathway	4843|4846|5294
53	Chemical carcinogenesis—receptor activation	154|196|5241|5970
54	Th17 cell differentiation	196|5530|5970
55	Chemical carcinogenesis—reactive oxygen species	196|847|5970
56	cAMP signaling pathway	135|154|1812|5970
57	NF‐kappa B signaling pathway	5328|5743|5970
58	MicroRNAs in cancer	5328|5743|7157
59	Diabetic cardiomyopathy	2539|4846|5970

### 3.3 Molecular Docking

**FIGURE 6:**
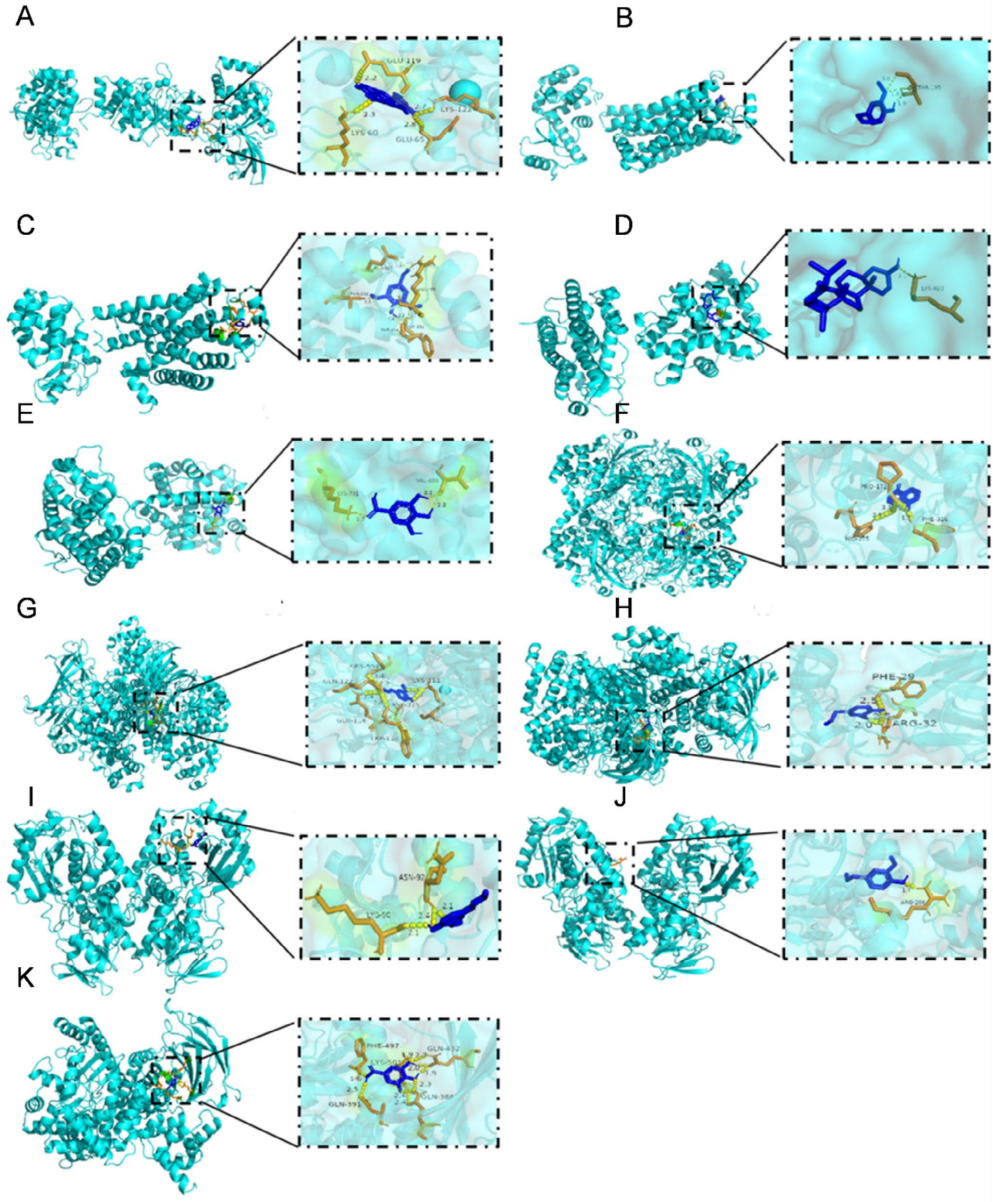
The sample graphs of molecular docking between walnut shell ingredients and the corresponding targets. (A) Molecular docking graph between PTPN2 and catechol; (B) Molecular docking graph between ADRB2 and eugenol; (C) Molecular docking graph between ADRB2 and syringaldehyde; (D) Molecular docking graph between PGR and campesterol; (E) Molecular docking graph between PGR and gallate; (F) Molecular docking graph between CAT and juglone; (G) Molecular docking graph between LTA4H and coniferaldehyde; (H) Molecular docking graph between LTA4H and eugenol; (I) Molecular docking graph between MAOA and coniferaldehyde; (J) Molecular docking graph between MAOA and eugenol; (K) Molecular docking graph between PIK3CG and gallate.

To validate the findings from network pharmacology studies, we selected key target proteins associated with the disease, specifically PTPN2, ADRB2, PGR, CAT, LTA4H, MAOA, and PIK3CG, and employed molecular docking with selected WSEE components. The results indicated that catechol, eugenol, eugenaldehyde, rapesosterol, gallic acid, walnut quinone, and coniferal exhibited strong binding affinities with their respective targets. The LYS‐60, GLU‐65, LYS‐122, and GLU‐119 residues of catechol formed four hydrogen bonds when docked with PTPN2, and the ΔG between catechol and PTPN2 was −8.4 kcal/mol. The THR‐195 residue of eugenol established three hydrogen bonds during its interaction with ADRB2, and the ΔG between eugenol and ADRB2 was −6.6 kcal/mol. Additionally, the PHE‐29 and ARG‐32 residues of eugenol created three hydrogen bonds upon docking with LTA4H, and the ΔG between eugenol and LTA4H was −5.8 kcal/mol. A single hydrogen bond was observed between the ARG‐26 residue of catechol and MAOA, and the ΔG between catechol and MAOA was −6.7 kcal/mol. Syringaldehyde's THR‐110, GLU‐107, ARG‐175, ASP‐192, and PHE‐194 residues engaged in six hydrogen bonds while interacting with ADRB2, and the ΔG between syringaldehyde and ADRB2 was −5.6 kcal/mol. The LYS‐822 residue of campesterol formed one hydrogen bond during its docking with PGR, and the ΔG between campesterol and PGR was −8.3 kcal/mol. The LYS‐731 and VAL‐698 residues of gallic acid established three hydrogen bonds in their interaction with PGR, the ΔG between which was −5.9 kcal/mol. The PHE‐497, GLN‐432, GLN‐388, GLN‐391, and LYS‐501 residues of gallic acid can dock with PIK3CG through nine hydrogen bonds, and the ΔG was −5.9 kcal/mol. The PRO‐172, HIS‐175, and PHE‐326 residues of walnut quinone can dock with CAT via four hydrogen bonds, and the ΔG between walnut quinone and CAT was −8 kcal/mol. Furthermore, the SER‐594, LYS‐111, ASP‐375, TRP‐117, GLN‐116, and GLN‐122 residues of coniferal can interact with LTA4H by forming six hydrogen bonds, and the ΔG between coniferal and LTA4H was −7 kcal/mol. Lastly, the ASN‐92 and LYS‐90 residues of eugenol are capable of establishing three hydrogen bonds when docking with MAOA, and the ΔG between MAOA and eugenol was −6.7 kcal/mol (Figure 6).

### 3.4 Experimental Verification

#### 3.4.1 Anti‐Inflammatory Effect of WSEE

**FIGURE 7:**
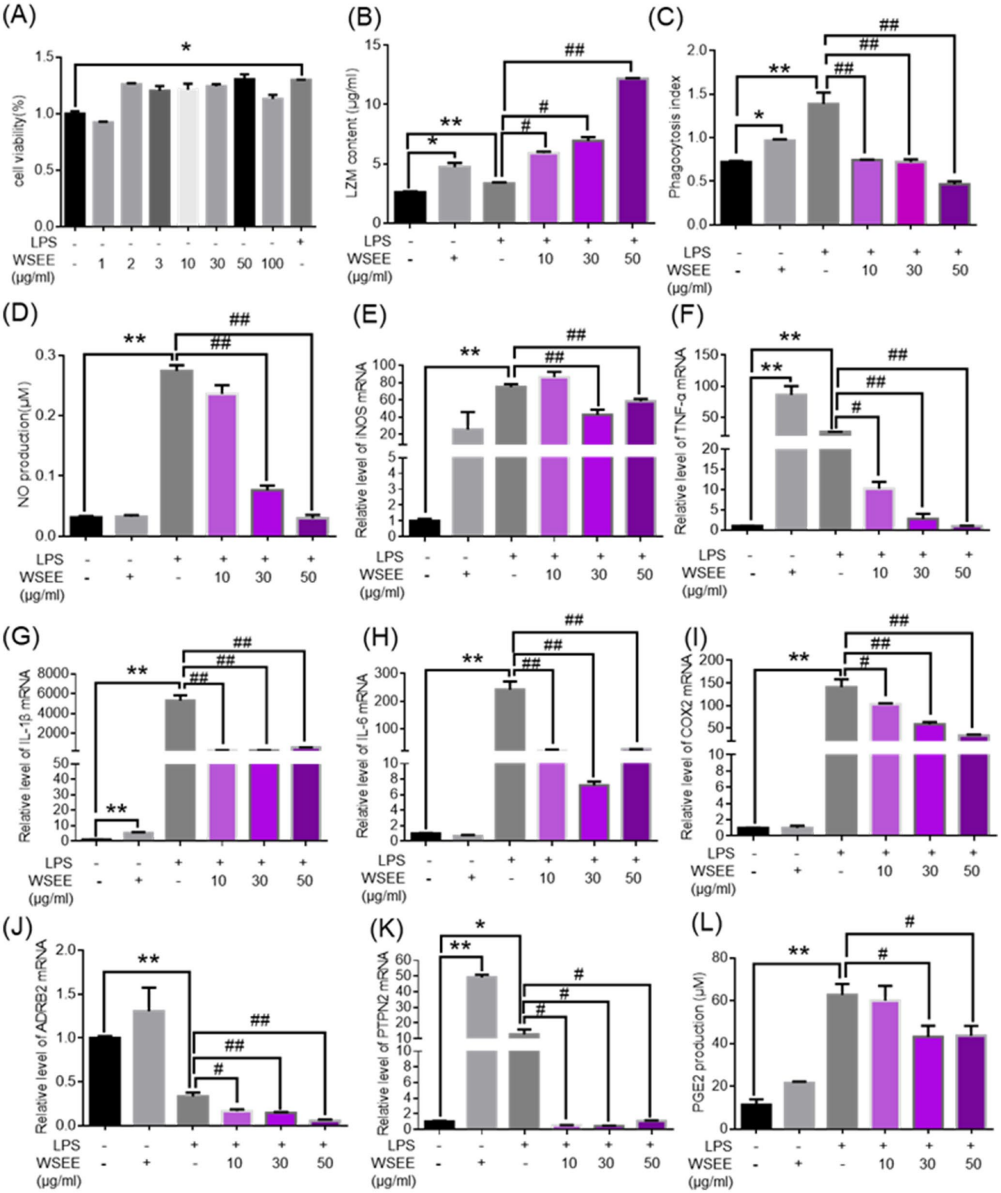
Anti‐inflammation effects on murine macrophage RAW264.7 of WSEE. (A) Cell viability; (B) LZM content; (C) phagocytic activity; (D) NO secretion; (E) the mRNA expression of iNOS; (F) TNF‐α; (G) IL‐1β; (H) IL‐6; (I) COX‐2; (J) ADRB2; (K) PTPN2; and (L) PGE2 secretion. *p < 0.05, **p < 0.01 vs. Con group; ^#^ *p* < *0.05*, ^##^ *p* < 0.01 vs. LPS group.

The cytotoxicity and proliferation of WSEE on RAW264.7 cells were evaluated using the MTT assay. As illustrated in Figure 7A, WSEE exhibited no cytotoxic effects on RAW264.7 cells at concentrations ranging from 1 to 100 μg/mL. The concentrations of 10, 30, and 50 μg/mL were used for the follow‐up experiments, among which, 30 μg/mL WSEE alone was designed as a control group.

Lipopolysaccharides (LPS) are well‐known inflammatory activators that can initiate various intercellular signaling pathways associated with inflammation (Björkbacka et al. 2004; Calvano et al. 2005; Gioannini et al. 2004). Lysozyme (LZM), an alkaline enzyme, hydrolyzes mucopolysaccharides found in pathogenic bacteria. Figure 7B demonstrates that WSEE can enhance LZM levels in macrophages and exhibit a synergistic effect on the LPS‐induced increase in LZM content in a concentration‐dependent manner.

Nitric oxide (NO) is recognized as a crucial inflammatory mediator involved in numerous pathological processes, including the induction or inhibition of apoptosis and immunoregulation. NO is synthesized by activated macrophages, which have been regarded as a quantitative index for assessing macrophage activation (MacMicking et al. 1997). Cyclooxygenase 2 (COX2) and inducible nitric oxide synthase (iNOS) are two key inflammatory enzymes that mediate inflammatory responses (Guzik et al. 2003; Kolb‐Bachofen 1998; MacMicking et al. 1997). Under normal conditions, iNOS and COX2 are minimally expressed. However, they can be induced by inflammatory stimuli to promote the production of inflammatory mediators such as NO and prostaglandin E2 (PGE2), thereby contributing to inflammation. LPS markedly enhanced phagocytosis (Figure 7C), NO production (Figure 7D), PGE2 production (Figure 7L), and the mRNA expression levels of iNOS, TNF‐α, IL‐1β, IL‐6, and COX‐2 (Figure 7E–I) within RAW264.7 cells (*p* < 0.01 for all). Conversely, WSEE effectively inhibited these LPS‐induced effects in a dose‐dependent manner. In summary, WSEE significantly mitigates the LPS‐induced inflammatory response, demonstrating strong anti‐inflammatory properties.

According to the components‐target network and the network's topological parameters, we identified key targets, including the β‐2 adrenergic receptor (ADRB2), leukotriene A4 hydrolase (LTA4H), and protein tyrosine phosphatase non‐receptor type 2 (PTPN2). ADRB2, which is associated with energy consumption and lipid metabolism (de Luis et al. 2023; Lafontan et al. 1997), serves as a target for catechol and eugenol. LPS reduced ADRB2 expression (*p* < 0.01) in RAW264.7 cells (*p* < 0.01). In contrast, WSEE significantly enhanced the transcriptional expression of ADRB2, and further combination treatment with WSEE and LPS resulted in an additional decrease in ADRB2 expression (*p* < 0.01).

Leukotriene A4 hydrolase (LTA4H) exhibits dual activity as both an aminopeptidase and an epoxide hydrolase. It is a key enzyme involved in the synthesis of leukotriene B4 (LTB4) and serves as a target for conic aldehyde and eugenol (Haeggstrom 1999; Low et al. 2017). LTB4 is produced by macrophages, neutrophils, and mast cells as an important downstream metabolite derived from unsaturated fatty acids through the 5‐lipoxygenase (5‐LOX) pathway (Lin et al. 2021; Xie et al. 2021). The binding of LTA4H to the leukotriene B4 (BLTR) receptor on cell membranes can activate granulocytes, leading to the generation of superoxide anions while also stimulating leukocyte chemotaxis, aggregation, the release of reactive oxygen species, lysosomal enzymes, increased vascular permeability, and constriction of blood vessel walls (Sanchez‐Galan et al. 2008; Yokomizo et al. 1997; Yokomizo et al. 2000). These physiological responses are believed to exacerbate post‐traumatic cellular injury. Notably, both WSEE and LPS alone significantly decreased the transcript expression levels of LTA4H (*p* < 0.01); however, when combined with LPS treatment, there was no significant effect on the low expression levels induced by LPS (Figure S1).

Protein tyrosine phosphatase nonreceptor type 2 (PTPN2) is a member of the protein tyrosine phosphatase family, which includes signaling molecules that regulate various cellular processes such as cell growth, differentiation, the mitotic cycle, and oncogenic transformation. Additionally, PTPN2 plays a role in growth factor‐mediated cell signaling (Jiang and Zhang 2008; Julien et al. 2010; Kuang et al. 2022). PTPN2 negatively regulates numerous biological processes, including hematopoiesis (Pike and Tremblay 2016; Tiganis and Bennett 2007), inflammatory response, cell proliferation and differentiation, and glucose homeostasis. It achieves this by dephosphorylating receptor protein tyrosine kinases as well as non‐receptor protein tyrosine kinases in both the nucleus and cytoplasm. Consequently, PTPN2 plays a multifaceted role that is crucial for the development of the immune system. WSEE or LPS alone significantly increased the transcription levels of PTPN2 in RAW264.7 cells (*p* < 0.01), while the combination of WSEE and LPS notably inhibited the expression induced by LPS. The Key molecules, including PGR, CAT, MAOA, and PIK3CG, have been identified through molecular docking studies; however, specific details and mechanisms remain to be elucidated.

#### 3.4.2 Procoagulation Effect of WSEE In Vitro and In Vivo

The coagulation process involves both endogenous (intrinsic) and exogenous (extrinsic) pathways. PT is indicative of the overall efficiency of the extrinsic pathway, while APTT correlates with the intrinsic pathway. TT measures the duration required for fibrinogen to convert into fibrin. Thrombin hydrolyzes fibrinogen to form insoluble fibrin, which promotes platelet aggregation and facilitates hemostasis. The results of the WSEE in vitro coagulation assay are presented in Table 5. Compared to the blank control group, both WSEE and Yunnan Bai Yao significantly shortened serum APTT, TT, and PT (*p < 0.01*), while also markedly increasing FIB levels (*p* < 0.01). Results from rat tail amputation hemostasis experiments demonstrated no significant difference in hemostasis times between the WSEE group and the positive control group receiving Yunnan Bai Yao, indicating a pronounced effect on promoting coagulation, as shown in Table 5. These findings suggest that WSEE activates both endogenous and exogenous coagulation pathways, and its efficacy is comparable to that of Yunnan Bai Yao.

**TABLE 5:** Effects of walnut shell components on coagulation in vitro and on hemostasis time of rat tail amputation (mean ± SD, *n* = 10; vs the normal saline group).

Group	APTT (s)	PT (s)	TT (s)	FIB (g/l)	Hemostasis times (s)
Normal saline	55.13 ± 1.6	18.63 ± 3.3	34.3 ± 3.2	1.87 ± 0.09	13:25
WSEE	40.4 ± 3.05**	14.6 ± 2.1**	26.13 ± 1.6*	3.7 ± 1.01**	05:26**
Yunnan Bai Yao	33.27 ± 3.01**	14.93 ± 1.6**	22.77 ± 2.3**	1.13 ± 0.63*	05:03**

* *p* < 0.05.

** *p* < 0.01.

#### 3.4.3 Skin Allergy Test and Acute Eye Allergy Test in Rats

**FIGURE 8:**
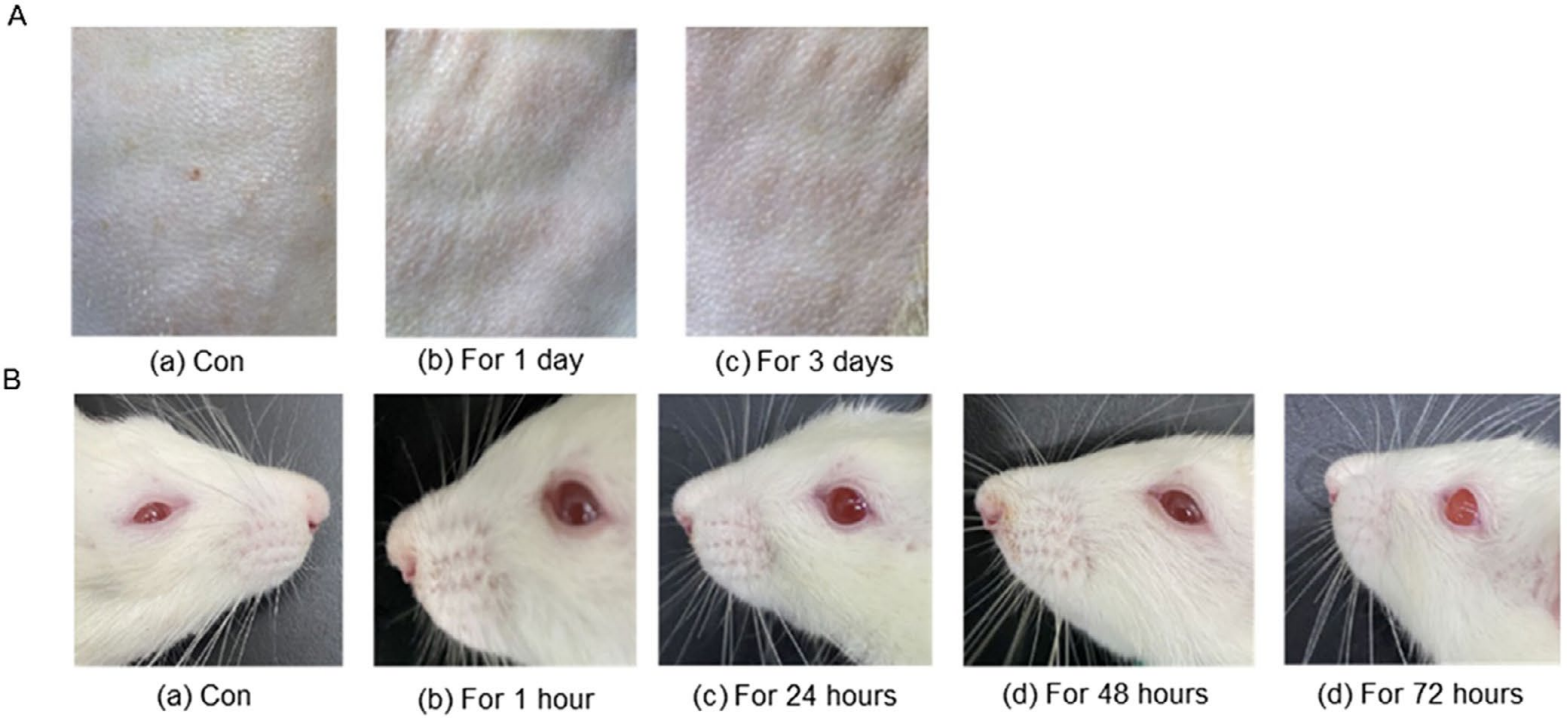
Skin allergy experiment (A) and acute eye allergy experiment (B) in rats.

Multiple allergy experiments on the eyes and skin of rats were conducted to assess the safety of WSEE for the human body (Di et al. 2022; Mehling et al. 2007; Ortega et al. 2016). WSEE was applied to the exposed skin of rats for 1 and 3 days, and no allergic reactions or damage, such as erythema or edema, were observed (Figure 8A). When WSEE was administered to the eyes of rats, the conjunctiva, iris, and cornea showed no abnormalities, and the rats did not exhibit any signs of discomfort within 72 h (Figure 8B). These findings indicate that WSEE has good biosafety as a wound dressing.

A vast range of suggested therapies for promoting wound healing, such as topical corticosteroids, antimicrobials, cyanoacrylate adhesives, and other immunomodulatory agents. However, the long‐term and repeated application of corticosteroids or antibiotics could cause adrenal suppression, such as thinning of skin (Lax et al. 2024), truncal obesity (Canapari et al. 2014), hypertension (Shen and Young 2012), and hyperglycemia (Haan et al. 2024). To circumvent the side effects of corticosteroids, the plant products are potential agents for wound healing because of their widespread availability, non‐toxicity, ease of administration, absence of unwanted side effects, and their effectiveness as crude preparations. For example, the clinical evaluations of wound healing showed a faster resolution of the healing in rats treated with chamomile than that observed in the group treated with corticosteroids (Martins et al. 2008); *Cynara humilis* is traditionally used for the management of wound healing and microbial infections (Ahmad et al. 2021); *Marantodes pumilum* has antioxidant effects that may enhance wound healing in the rat model (Ahmad et al. 2021).

With minimal side effects and great potential for wound healing, WSEE has been preliminarily studied; however, there are two potential issues in this study that need to be noted. Firstly, animal models for medical and biological research are an important foundation of medical research. However, there are significant limitations and challenges when the results obtained from such studies are applied to clinical applications. The limitations of animal models in the study are as follows: Firstly, differences between the experimental environment and the real situation in humans; secondly, biological differences between species should be taken into consideration, such as physiological and anatomical differences, pathological differences of disease, and differences in drug metabolism and reactions. The challenges of applying the results of animal research to humans are as follows: Animal models cannot reflect the treatment effect of individual differences within human populations due to factors such as age, gender, genetic background, lifestyle, and comorbidities; a large number of drugs or therapies that have shown effectiveness in animal models, however, would fail when they enter human clinical trials (especially in Phase II/III), which is mainly due to species differences, inaccurate disease models, and limitations of preclinical research; (Widerspick et al. 2023). How to convert the effective dose/toxic dose obtained from animal models to the human body accurately and safely is also complex and risky.

In addition, as a natural product, WSEE is not absolutely safe. Their potential toxicological risks are complex and diverse due to the inherent toxic components, contamination and adulteration, improper use, and drug interactions, among other factors. There is an urgent and significant necessity to conduct systematic, standardized, and in‐depth toxicity studies. Future research should focus on the basic toxicity data and delve deeper into specific toxicities and mechanisms of WSEE.

## 4 Conclusion

In summary, this study elucidated the primary active ingredients of WSEE. The main targets associated with promoting wound healing were identified as PTPN2, ADRB2, PGR, CAT, LTA4H, MAOA, and PIK3CG. WSEE exhibited synergistic anti‐inflammatory and coagulation pharmacological effects through the cGMP‐PKG signaling pathway, along with IL‐17, cAMP, and NF‐κB. Moreover, the potential molecular mechanisms of WSEE were validated in vivo and in vitro experiments, which indicated that WSEE has good procoagulation and anti‐inflammatory characteristics.

The above results hinted at the potential of WSEE in hemostasis and wound healing promotion and broadened the possibility of its clinical application. Future research needs to clarify the key mechanism of WSEE in regulating wound healing through metabolomics and proteomics, providing the theoretical basis for its clinical application. In terms of clinical application, WSEE can be prepared as a composite dressing with good biological compatibility and antibacterial effect. For example, its anti‐inflammatory properties can be used for the management of chronic wounds such as diabetic foot and pressure ulcers; surgical dressings with WSEE can reduce the risk of bleeding and infection; it can also be used for rapid healing of superficial injuries such as burns and abrasions. However, there are still some challenges in its industrialization and clinical transformation. For example, the standardized production needs to ensure the consistency of the batch and clarify the efficacy threshold of the active ingredient; the toxicity data of long‐term use needs to be further supplemented, especially the impact on the immune system; enzymatic hydrolysis and freezing processes may increase production costs and need to be optimized.

## Author Contributions

**Ying He:** data curation (lead), formal analysis (equal), investigation (lead), methodology (lead), software (equal), validation (lead), writing – original draft (lead). **Wenhui Zhao:** methodology (supporting), software (equal), writing – review and editing (supporting). **Yanting Sun:** methodology (supporting), software (equal). **Lei Ma:** investigation (equal), software (equal). **Censhu Li:** software (equal). **Xueying Zheng:** investigation (equal). **Zeguo Feng:** methodology (equal), software (supporting). **Hui Guo:** supervision (supporting). **Liguo Qin:** supervision (lead). **Yali Zhang:** funding acquisition (equal), project administration (equal), supervision (lead), visualization (equal), writing – review and editing (equal).

## Conflicts of Interest

The authors declare no conflicts of interest.

## Supporting information


Data S1.

## Data Availability

The original contributions presented in the study are included in the article, further inquiries can be directed to the corresponding author.
