# Supplementary material for: Elucidation of the Hemostatic and Anti‐Inflammatory Effects of Walnut Shell Ethanol Extract by Network Pharmacology and Experimental Verification
Source: Food Sci Nutr. 2025 Aug 5;13(8):e70726. doi: 10.1002/fsn3.70726 (PMC12325102; doi:10.1002/fsn3.70726)
Supplement: Supplementary file 1 — Data S1. [file FSN3-13-e70726-s001.docx]

**Supporting Information**

**Elucidation of the Hemostatic and Anti-inflammatory Effects of Walnut Shell Ethanol Extract by** **Network Pharmacology and experimental verification**

Ying He ^a^, Wenhui Zhao ^b^, Censhu Li^a^, Xueying Zheng^a^, Zeguo Feng^a^, Yanting Sun^a^, Lei Ma^a^, Hui Guo ^c^, Liguo Qin ^b,^ *, Yali Zhang ^a,^ *

^a^ Center for Mitochondrial Biology and Medicine, The Key Laboratory of Biomedical Information Engineering of Ministry of Education, School of Life Science, Xi’an Jiao tong University, Xi’an 710049, China

^b^ Key Laboratory of Education Ministry for Modern Design and Rotor-Bearing System, Institute of Design Science and Basic Components. School of Mechanical Engineering, Xi’an Jiao tong University, Xi’an, 710049, P.R. China

^c^ Department of Endocrinology, First Affiliated Hospital of Medical College, Xi'an Jiao tong University, Xi'an, 710061, China

*Corresponding author: Dr. Yali Zhang, [yar.lee@mail.xjtu.edu.cn](mailto:yar.lee@mail.xjtu.edu.cn).

Table S1 Chemical Constituents of WSEE

| NO. | Compound name | CAS NO. | Molecular formula | Relative molecular mass | *m/z* [M-H] | t_R_/min | Relative Abundance (%) |
| --- | --- | --- | --- | --- | --- | --- | --- |
| 1 | 2,4-Dihydroxy-2,5-dimethyl-3(2H)-furan-3-one | 10230-62-3 | C_6_H_8_O_4_ | 144.1253 | 162.0763 | 0.31 | 0.199403 |
| 2 | Paeonoside A | — | C_14_H_18_O_9_ | 330.2873 | 353.0841 | 0.33 | 2.99104 |
| 3 | 6' -o-vanillin tacoside | — | C_21_H_24_O_11_ | 452.409 | 491.0971 | 0.34 | 0.411161 |
| 4 | Dihydrocarthiopanate -4 '-O-β -D-glucopyranoside | — | C_21_H_32_O_10_ | 444.473 | 483.1635 | 0.34 | 6.308555 |
| 5 | Roseoside | 54835-70-0 | C_19_H_30_O_8_ | 386.4370 | 409.1861  (387.203） | 0.34 | 1.565683 |
| 6 | 1-(Carboxymethyl)Piperidine-4-Carboxylic Acid | 53919-19-0 | C_8_H_13_NO_4_ | 187.193 | 188.0919 | 0.35 | 0.343248 |
| 7 | 5-Hydroxymethyl-2-furaldehyde | 67-47-0 | C_6_H_6_O_3_ | 126.11 | 127.0392 | 0.37 | 0.152191 |
| 8 | Juglone | 481-39-0 | C_10_H_6_O_3_ | 174.1528 | 192.0653（173.02） | 0.47 | 0.086732 |
| 9 | 3-Hydroxycyclohexanone | 823-19-8 | C_6_H_10_O_2_ | 114.1424 | 132.1022 | 0.53 | 0.361658 |
| 10 | 4-Hydroxybenzoic Acid | 99-96-7 | C_7_H_6_O_3_ | 138.1207 | 139.0391 | 0.89 | 1.488361 |
| 11 | 1-Formyl-2-Piperidinecarboxylic Acid | 54966-20-0 | C_7_H_11_NO_3_ | 157.167 | 158.0814 | 1.38 | 0.089187 |
| 12 | 6-Cyclohexyl-4-Methyl-2H-Pyran-2-One | 14818-35-0 | C_12_H_16_O_2_ | 192.2542 | 119.0494 | 1.47 | 0.317882 |
| 13 | 4'-Hydroxy-3'-Methoxyacetophenone | 498-02-2 | C_9_H_10_O_3_ | 166.1739 | 167.0703 | 1.53 | 1.255165 |
| 14 | 3-Hydroxy-4-methoxy-benzoic acid | 645-08-9 | C_8_H_8_O_4_ | 168.1467 | 169.0497 | 1.65 | 1.988299 |
| 15 | Ethyl Maltol | 4940-11-8 | C_7_H_8_O_3_ | 140.1366 | 141.0547 | 1.68 | 0.331792 |
| 16 | 3-hydroxy-1-(4-hydroxy-3-methoxyphenyl)propan-1-one | 2196-18-1 | C_10_H_12_O_4_ | 196.19988 | 197.0809 | 1.76 | 1.600049 |
| 17 | 4-Hydroxybenzaldehyde | 123-08-0 | C_7_H_6_O_2_ | 122.1213 | 123.0443 | 1.9 | 1.901158 |
| 18 | O-isopropyl cyclohexanol | 96-07-1 | C_9_H_18_O | 142.2386 | 165.1274 | 1.98 | 0.164464 |
| 19 | 4-Allylphenol | 501-92-8 | C_9_H_10_O | 134.1751 | 135.0806 | 2 | 0.253651 |
| 20 | Juglanoside A | — | C_16_H_20_O_7_ | 324.326 | 347.1126 | 2.1 | 3.278648 |
| 21 | Methyl Vanillate | 3943-74-6 | C_9_H_10_O_4_ | 182.1733 | 183.0651 | 2.26 | 0.407479 |
| 22 | 5-Ethyl-2-Furaldehyde | 23074-10-4 | C_7_H_8_O_2_ | 124.137 | 125.0598 | 2.27 | 0.858323 |
| 23 | 3-Hydroxy-5-Methoxybenzaldehyde | 57179-35-8 | C_8_H_8_O_3_ | 152.1473 | 153.0547 | 2.28 | 0.998241 |
| 24 | Ethyl Vanillate | 617-05-0 | C_10_H_12_O_4_ | 196.1999 | 197.081 | 2.4 | 2.205539 |
| 25 | Syringealdehyde | 134-96-3 | C_9_H_10_O_4_ | 182.1733 | 183.0652 | 2.42 | 0.659494 |
| 26 | 1- (4 '-hydroxyphenyl) -allyl aldehyde | 2538-87-6、20711-53-9 | C_9_H_8_O_2_ | 148.1586 | 149.06 | 3.06 | 4.577589 |
| 27 | Coniferaldehyde | 458-36-6、20649-42-7 | C_10_H_10_O_3_ | 178.1846 | 179.0702 | 3.08 | 13.01559 |
| 28 | 4-Hydroxy-3-Methoxyphenylacetone | 2503-46-0 | C_10_H_12_O_3_ | 180.200 | 181.0853 | 3.08 | 0.583807 |
| 29 | Guaiacol | 90-05-1 | C_7_H_8_O_2_ | 124.1372 | 147.0443（125.0） | 3.08 | 2.964857 |
| 30 | Juglanoside E | — | C_16_H_20_O_9_ | 356.3246 | 395.0765 | 3.13 | 0.153418 |
| 31 | 1,2-Diphenylcyclobutane | 3018-21-1 | C_16_H_16_ | 208.2982 | 105.0702 | 3.24 | 0.49953 |
| 32 | 3-(2,5-Dimethoxyphenyl)propionic acid | 10538-49-5 | C_11_H_14_O_4_ | 210.2265 | 211.0962 | 3.61 | 2.170765 |
| 33 | 2-Methoxy-4-Vinylphenol | 7786-61-0 | C_9_H_10_O_2_ | 150.1745 | 151.0753 | 3.68 | 1.759604 |
| 34 | N-Octylmalonic Acid | 760-55-4 | C_11_H_20_O_4_ | 216.2741 | 255.1013 | 3.7 | 3.072045 |
| 35 | 2- aldehyde -3- hydroxyphenylpropanol | — | C_9_H_10_O_3_ | 166.1739 | 167.0702 | 3.72 | 10.69631 |
| 36 | Phenol acetate | 122-79-2 | C_8_H_8_O_2_ | 136.15 | 137.0598 | 3.83 | 9.820398 |
| 37 | Eugenol | 97-53-0 | C_10_H_12_O_2_ | 164.2011 | 187.0756 | 4.07 | 0.898008 |
| 38 | Campestrol | 4651-51-8 | C_28_H_48_O | 400.6801 | 439.3312 | 6.03 | 2.374095 |
| 39 | beta-Sitosterol | 83-46-5 | C_29_H_50_O | 414.7067 | 437.3732 | 7.02 | 0.149327 |
| 40 | 3-Tert-Butyl-4-Hydroxyanisole | 121-00-6、25013-16-5 | C_11_H_16_O_2_ | 180.2435 | 203.1067 | 7.53 | 0.846868 |
| 41 | Pyrogallol | 87-66-1 | C_6_H_6_O_3_ | 126.11 | 149.023 | 8.03 | 2.722252 |
| 42 | 9,11-Octadecadienoic Acid | 13058-52-1 | C_19_H_34_O_2_ | 294.4720 | 317.2475 | 14.04 | 0.25897 |
| 43 | 4-Stigmasten-3-One | 1058-61-3 | C_29_H_48_O | 412.6908 | 413.3776 | 17 | 11.50473 |
| 44 | Caulophyllogenin | 52936-64-8 | C_30_H_48_O_5_ | 488.699 | 489.3574 | 17 | 0.377613 |
| 45 | Vinyl propionate | 105-38-4 | C_5_H_8_O_2_ | 100.11 | 118.0865 | 20.84 | 0.640674 |


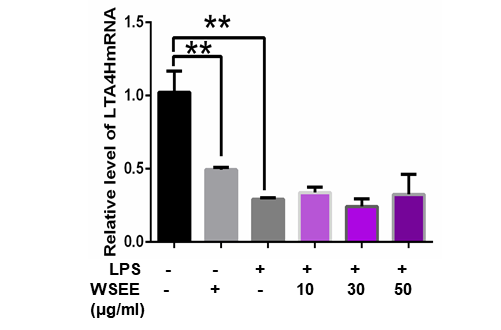
Fig. S1 Anti-inflammation effects on murine macrophage RAW264.7 of WSEE. The mRNA expression of LTA4H.

The primer sequence for LTA4H:

Forward: GTCCCGAAAGAACTGGTG

Reverse: CCA AAGCAATCAGGTAGCA
